# Charge balanced control of seizure like activity in a two dimensional cortical model

**DOI:** 10.1186/1471-2202-13-S1-P33

**Published:** 2012-07-16

**Authors:** Prashanth Selvaraj, Andrew Szeri

**Affiliations:** 1 Department of Mechanical Engineering, University of California, Berkeley, CA 94703, USA; 2 Center for Neural Engineering and Prosthesis, UC Berkeley and UC San Francisco, USA

## 

The invasiveness of surgical procedures and the ineffectiveness of medication in remedying seizures in some epileptic patients have led to investigations into alternative methods such as feedback control. While the methods for applying control are varied, the underlying idea is to use electrical signals to disrupt irregular cortical activity. Experimentally, an adaptively applied radial electric field has been shown to control seizure like activity in a mammalian brain [1].

In previous work, a meso scale model consisting of stochastic partial differential equations was developed [2]. Seizure like activity has been simulated with this model, and successfully suppressed using charge balanced electrical control [3]. The magnitude of control was related to the signal sensed at the cortical surface. This work extends previous research to a *two dimensional* meso scale cortical model. Cortical waves in a two dimensional spatial domain present more complex dynamics than are seen in the one dimensional model, with a greater variety of instabilities of the basic (healthy) states. This presents greater challenges for successful control.
